# Efficacy and safety of the combination of encorafenib/cetuximab with or without binimetinib in patients with *BRAF* V600E-mutated metastatic colorectal cancer: an AGEO real-world multicenter study

**DOI:** 10.1016/j.esmoop.2024.103696

**Published:** 2024-09-09

**Authors:** C. Gallois, E.S. Bergen, É. Auclin, S. Pernot, J. Higué, I. Trouilloud, Y. Touchefeu, A. Turpin, T. Mazard, A. Sartore-Bianchi, H. Prenen, A. Alberti, L. Pilla, S. Cuissy, V. Wookey, A. Perret, C. Melchior, P. Artru, O. Dubreuil, A. Drouillard, S. Doat, J. Lavolé, D. Basile, G. Perkins, M. Jary, S. Stintzing, J. Jos, D. Tougeron, J. Taieb

**Affiliations:** 1 Department of Gastroenterology and Digestive Oncology, Paris-Cité University, Georges Pompidou European Hospital, SIRIC CARPEM, Paris, France; 2 Division of Oncology, Department of Medicine I, Medical University of Vienna, Vienna, Austria; 3 Medical and Thoracic Oncology Department, Hôpital Européen Georges Pompidou, AP-HP, Paris, France; 4 Department of Medical Oncology, Institut Bergonié, Bordeaux, France; 5 Centre Hospitalier Universitaire de Toulouse, Toulouse, France; 6 Department of Medical Oncology, Hôpital Saint-Antoine, AP-HP, Paris, France; 7 Digestive Oncology, Institut Des Maladies De l’Appareil Digestif, Centre Hospitalier Universitaire De Nantes, Nantes, France; 8 Department of Medical Oncology, University Lille, Lille, France; 9 UMR9020 CNRS, UMR-S1277 Inserm, Canther - Cancer Heterogeneity, Plasticity and Resistance to Therapies, CHU Lille, Lille, France; 10 Department of Medical Oncology, Montpellier Cancer Institute (ICM), Montpellier, France; 11 Institut de Recherche en Cancérologie de Montpellier (IRCM), INSERM U1194, University of Montpellier, Montpellier, France; 12 Department of Oncology and Hemato-Oncology, Università degli Studi di Milano and Niguarda Cancer Center, Grande Ospedale Metropolitano Niguarda, Milan, Italy; 13 University Hospital Antwerp, Edegem, Belgium; 14 Medical Oncology, University of Brescia, ASST-Spedali Civili, Brescia, Italy; 15 Department of Hepatogastroenterology, Rouen University Hospital, Rouen, France; 16 Department of Oncology, Mayo Clinic, Rochester, USA; 17 Department of Medical Oncology, Gustave Roussy Cancer Centre, Villejuif, France; 18 Department of Medical Oncology, Centre Léon Bérard, Lyon, France; 19 Hepatogastroenterology Department, Hôpital Jean-Mermoz, Lyon, France; 20 Department of Digestive Oncology, Groupe hospitalier Diaconesses Croix Saint Simon, Paris, France; 21 Department of Hepato-Gastroenterology, Dijon Hospital, Dijon, France; 22 Department of Hepato-Gastroenterology, Pitié-Salpêtrière Hospital, Paris, France; 23 Department of Hepato-Gastroenterology, Begin Teaching Military Hospital, Saint-Mandé, France; 24 Department of Medical Oncology, San Giovanni di Dio Hospital, Crotone, Italy; 25 Department of Gastroenterology, CHRU Pontchaillou, Rennes, France; 26 Department of Surgical and Medical Oncology, University Hospital of Clermont-Ferrand, Clermont-Ferrand, France; 27 Department of Hematology, Oncology, and Cancer Immunology (CCM), Charité-Universitätsmedizin Berlin, Berlin, Germany; 28 Department of Medical Oncology, Vall d’Hebron University Hospital, Barcelona, Spain; 29 Department of Gastroenterology and Hepatology, Poitiers University Hospital, Poitiers, France

**Keywords:** colorectal cancer, *BRAF* V600E mutation, encorafenib, targeted therapy, real-world study

## Abstract

**Background:**

The combination of encorafenib with cetuximab has become the standard of care in patients with *BRAF* V600E-mutated metastatic colorectal cancer (mCRC) after a prior systemic therapy. This study aims to describe the efficacy and safety of encorafenib/cetuximab +/− binimetinib in patients with *BRAF* V600E-mutated mCRC in a real-world setting.

**Patients and methods:**

This retrospective study included patients with *BRAF* V600E-mutated mCRC who received this combination from January 2020 to June 2022 in 30 centers.

**Results:**

A total of 201 patients were included, with 55% of women, a median age of 62 years, and an Eastern Cooperative Oncology Group performance status (ECOG-PS) >1 in 20% of cases. The main tumor characteristics were 60% of right-sided primary tumor, 11% of microsatellite instability/mismatch repair deficient phenotype, and liver and peritoneum being the two main metastatic sites (57% and 51%). Encorafenib/cetuximab +/− binimetinib was prescribed in the first, second, third, and beyond third line in 4%, 56%, 29%, and 11%, respectively, of cases, with the encorafenib/cetuximab/binimetinib combination for 21 patients (10%). With encorafenib/cetuximab treatment, 21% of patients experienced grade ≥3 adverse events (AEs), with each type of grade ≥3 AE observed in <5% of patients. The objective response rate was 32.2% and the disease control rate (DCR) was 71.2%. The median progression-free survival (PFS) was 4.5 months [95% confidence interval (CI) 3.9-5.4 months] and the median overall survival (OS) was 9.2 months (95% CI 7.8-10.8 months). In multivariable analysis, factors associated with a shorter PFS were synchronous metastases [hazard ratio (HR) 1.66, *P* = 0.04] and ECOG-PS >1 (HR 1.88, *P* = 0.007), and those associated with a shorter OS were the same factors (HR 1.71, *P* = 0.03 and HR 2.36, *P* < 0.001, respectively) in addition to treatment beyond the second line (HR 1.74, *P* = 0.003) and high carcinoembryonic antigen level (HR 1.72, *P* = 0.003).

**Conclusion:**

This real-world study showed that in patients with *BRAF* V600E-mutated mCRC treated with encorafenib/cetuximab +/− binimetinib, efficacy and safety data confirm those reported in the BEACON registration trial. The main poor prognostic factors for this treatment are synchronous metastases and ECOG-PS >1.

## Highlights

• This is a real-world study of BRAF V600E-mutated metastatic CRC patients treated with encorafenib/cetuximab +/− binimetinib.
• The tolerance profile was good with only 21% of grade ≥3 AEs.
• The efficacy data confirmed those reported in the BEACON CRC trial.
• The major prognostic factors for this treatment were synchronous metastases and ECOG-PS >1.

## Introduction

Molecular profiling of tumors in oncology has improved patient management by providing better prognostic stratification and, especially for certain molecular alterations, the advent of new targeted therapies. Thus, ∼10% of metastatic colorectal cancer (mCRC) harbor a *BRAF* V600E mutation, associated with a poor prognosis and a limited response to systemic historical treatments in mCRC.^1^^,^^2^

Encorafenib is a highly specific competitive inhibitor of RAF that acts solely on tumor cells expressing the BRAF V600E-mutated protein, exhibiting a more prolonged pharmacodynamic activity compared with other BRAF inhibitors.^3^ While the efficacy of BRAF inhibitors has been demonstrated in other cancers such as melanoma^4^ and non-small-cell lung cancer,^5^ their effectiveness remains limited as monotherapy for *BRAF* V600E-mutant mCRC,^6^ due to reactivation of the mitogen-activated protein (MAP) kinase signaling pathway by other RAF proteins such as CRAF and epidermal growth factor receptor (EGFR) overexpression.^7^ Thus the combination of a BRAF inhibitor with an EGFR monoclonal antibody enhances treatment efficacy in patients with *BRAF* V600E mCRC.^8^, ^9^, ^10^ The phase III BEACON CRC trial, involving 665 patients with *BRAF* V600E mCRC in the second or third line of treatment, demonstrated that the combination of encorafenib and cetuximab with or without binimetinib significantly improved objective response rate (ORR) and overall survival (OS) compared with standard of care (irinotecan-based chemotherapy plus cetuximab), with an acceptable safety profile.^11^ Although the two experimental arms cannot be directly compared due to the study design, the updated results of this trial showed a better ORR of 26.8% in patients treated with the triplet compared with 19.5% in patients treated with the doublet-targeted agents’ combination; however, this did not translate into a prolonged progression-free survival (PFS) or OS. In addition, the rate of grade ≥3 adverse events (AEs) was higher in patients treated with the triplet encorafenib/cetuximab/binimetinib (65.8%) compared with those treated with the doublet encorafenib/cetuximab (57.4%). For all these reasons, only the combination of encorafenib and cetuximab was approved in 2020 as the new standard treatment in mCRC with *BRAF* V600E mutation following prior systemic therapy^12^ in the second or third line of treatment by the European Medicines Agency, United States Food and Drug Administration, and other regulatory agencies.

After demonstrating the efficacy of a new treatment in a randomized trial, real-world data become essential to analyze its efficacy and tolerability in all-comer patients, who may not necessarily meet the eligibility criteria of the registration trial (for example, due to advanced age, comorbidities, poor overall health status). This brings additional information and helps to position the new treatment within the global therapeutic strategy for these patients. Three real-world studies have been published very recently with sample sizes ranging from 81 to 166 patients and a median follow-up that is still relatively short given the recent approval of this combination, ranging from 9.7 months to 14.5 months.^13^, ^14^, ^15^

Our objective was to include a larger real-world population with a longer follow-up period, to assess the long-term efficacy, the factors associated with shorter survival, the data on subsequent lines, and the safety of the combination encorafenib and cetuximab with or without binimetinib in patients with *BRAF* V600E-mutated mCRC.

## Patients and methods

### Patients

This retrospective, multicenter study involved 30 centers, including 22 French centers and 8 non-French centers (Italy, the United States, Spain, Germany, Belgium, and Austria). All consecutive patients with histologically confirmed *BRAF* V600E-mutated mCRC having received encorafenib plus cetuximab +/− binimetinib from January 2020 to June 2022 were included, with patient identification by the medical information systems of each center. Patients treated for *BRAF* non-V600E-mutated mCRC were excluded.

### Data collection

Data collected comprised a history of adjuvant treatments if any, baseline clinicopathological characteristics, and the different treatment sequences. The data received for each patient were centrally reviewed to define the lines of treatment with the same definition for the entire cohort, as described by Saini and Twelves.^16^ In addition, in patients with disease progression during or within 6 months after the end of adjuvant chemotherapy for localized CRC, this adjuvant regimen was considered as the first line of treatment for the metastatic disease, with the metastatic disease classified as synchronous.

For encorafenib/cetuximab +/− binimetinib therapy, the following were collected: graded AEs, reductions in treatment doses or discontinuation for toxicity, the date of surgery or ablation of metastases if any, response rate [ORR and disease control rate (DCR)] and primary progression rate evaluated as part of the standard of care, date of last news, and date of death.

Investigators retrospectively collected data on all consecutively enrolled patients from electronic medical records after obtaining their informed consent. A waiver of consent was considered for deceased patients or those lost to follow-up. The study was conducted in accordance with the Declaration of Helsinki and was approved by the ethical review board of the coordinating center CERAPHP Centre (IRB registration number 0001 1928).

### Statistical analyses

The median (interquartile range) values and proportions (percentage) were used for continuous and categorical variables, respectively. The median and proportions were compared using the Wilcoxon–Mann–Whitney test and the chi-square test (or Fisher’s exact test, if appropriate), respectively.

PFS was defined as the time between the start of encorafenib/cetuximab +/− binimetinib and tumor progression or death, whichever occurred first. OS was defined as the time between encorafenib/cetuximab +/− binimetinib start and death from any cause. Patients known to be alive were censored at the date of their last follow-up.

PFS and OS were estimated using the Kaplan–Meier method and described using the median or rate at specific time points with their 95% confidence intervals (CIs). Follow-up was calculated using the reverse Kaplan–Meier method.

The factors associated with PFS and OS were investigated using univariate and multivariable Cox models. The adjustment factors used in the multivariable analyses for PFS and OS were the variables with a *P* value <0.10 in the univariate analyses or clinically pertinent variables (such as line of treatment), with a limit of one adjustment variable for 10 events.

All analyses were carried out using R software version 2.15.2 (R Foundation, Vienna, Austria; https://www.r-project.org). *P* values of <0.05 were considered statistically significant, and all tests were two-sided.

## Results

### Patient characteristics

A total of 201 patients with mCRC diagnosed with *BRAF* V600E-mutated mCRC and treated with encorafenib/cetuximab +/− binimetinib were included, comprising 180 patients (89.5%) treated with the doublet encorafenib/cetuximab and 21 patients (10.4%) treated with the triplet encorafenib/cetuximab/binimetinib in specialized and/or academic centers in the majority of cases (91%). The median age was 62 years (range 29-90 years), 44.5% were men, and 19.9% had three or more metastatic sites. The primary tumor was frequently located on the proximal colon (59.8%), and the two most common metastatic sites were the liver and the peritoneum (57.2% and 50.7%, respectively; Table 1).

**Table 1:** Patient’s characteristics

Characteristics			Whole population (*N* = 201)	Encorafenib/cetuximab (*n* = 180), *n* (%)	Encorafenib/cetuximab/binimetinib (*n* = 21), *n* (%)
**Countries, *n* (%)**	France		134 (66.7)	124 (68.9)	10 (47.6)
	Spain		23 (11.4)	23 (12.8)	0
	Austria		17 (8.5)	7 (3.9)	10 (47.6)
	United States		10 (5)	10 (5.6)	0
	Italy		9 (4.5)	9 (5)	0
	Germany		5 (2.5)	4 (2.2)	1 (4.8)
	Belgium		3 (1.5)	3 (1.7)	0
**Treated in specialized and/or academic center, *n* (%)**	Yes		183 (91.0)	165 (91.7)	18 (85.7)
	No		18 (9.0)	15 (8.3)	3 (14.3)
**Sex**	Male, *n* (%)		89 (44.5)	77 (43)	12 (57.1)
	Female, *n* (%)		111 (55.5)	102 (57)	9 (42.9)
	Missing, *n*		1	1	0
**Age, years**	Median (range)		62 (29-90)	62.5 (31-90)	61 (29-82)
**Time to metastasis, *n* (%)**	Synchronous		164 (81.6)	148 (82.2)	16 (76.2)
	Metachronous		37 (18.4)	32 (17.8)	5 (23.8)
**Surgery of the primary tumor, *n* (%)**			141 (70.1)	128 (71.1)	13 (61.9)
**Primary tumor location**	Proximal		119 (59.8)	108 (60.7)	11 (52.4)
	Distal		54 (27.1)	47 (26.4)	7 (33.3)
	Rectum		26 (13.1)	23 (12.9)	3 (14.3)
	Missing, *n*		2	2	0
**Metastatic sites**	Liver		115 (57.2)	100 (55.6)	15 (71.4)
	Peritoneum		102 (50.7)	94 (52.2)	8 (38.1)
	Lung		46 (22.9)	43 (23.9)	3 (14.3)
	Lymph node		50 (24.9)	44 (24.4)	6 (28.6)
	Ovary		8 (4)	7 (3.9)	1 (4.8)
	Skin		6 (3)	4 (2.2)	2 (9.5)
**Number of metastatic sites ≥3, *n* (%)**			40 (19.9)	36 (20)	4 (19)
**Differentiation**	Well		31 (18.9)	30 (20.5)	1 (5.6)
	Moderate		80 (48.8)	72 (49.3)	8 (44.4)
	Poor		53 (32.3)	44 (30.1)	9 (50)
	Missing, *n*		37	34	3
**RAS mutated, *n* (%)**			4 (2)	2 (1.1)	2 (10)
**dMMR and/or MSI-high, *n* (%)**			23 (11.4)	22 (12.2)	1 (4.8)
**Prior oxaliplatin, *n* (%)**			164 (81.6)	149 (82.8)	15 (71.4)
**Prior irinotecan, *n* (%)**			143 (71.1)	130 (72.2)	13 (61.9)
**Prior bevacizumab/aflibercept, *n* (%)**			143 (71.1)	129 (71.7)	14 (66.7)
**Priori anti-EGFR, *n* (%)**			24 (11.9)	21 (11.7)	3 (14.3)
**Prior ICI, *n* (%)**			21 (10.4)	20 (11.1)	1 (4.8)
**Characteristics of encorafenib/cetuximab +/− binimetinib treatment, *n* (%)**	**Number line**	L1	8 (4)	3 (1.7)	5 (23.8)
		L2	113 (56.2)	104 (5.8)	9 (42.9)
		L3	58 (28.9)	54 (30)	4 (19)
		>L3	22 (10.9)	19 (10.5)	3 (14.3)
	**Baseline ECOG-PS**	0-1	157 (79.7)	139 (79)	18 (85.7)
		>1	40 (20.3)	37 (21)	3 (14.3)
		Missing, *n*	4	4	0
	**Baseline CEA**	Median (range)	14 (0-10,631)	14.4 (0-10,631)	23 (0.9-4429)
	**Cetuximab administration schedule, *n* (%)**	1 week	123 (61.2)	111 (61.7)	12 (57.1)
		2 weeks	78 (38.8)	69 (38.3)	9 (42.9)
**Subsequent lines, *n* (%)**			99 (46.3)	82 (45.5)	17 (80.9)

CEA, carcinoembryonic antigen; dMMR, deficient mismatch repair; ECOG-PS, Eastern Cooperative Oncology Group performance status; EGFR, epidermal growth factor receptor; ICI, immune checkpoint inhibitor; MSI, microsatellite instability.

The *BRAF* mutational status was determined based on a next-generation sequencing panel in 56.7% of cases, on a targeted multiplex PCR in 33.8% of cases, on circulating tumor DNA in 2.0% of cases, and with an unknown or another method in 7.5% of cases. Only 23 patients (11.4%) had a microsatellite instability (MSI)-high/mismatch repair deficient (dMMR) tumor determined by PCR and/or immunohistochemistry.

Among the 63 patients who received adjuvant chemotherapy after the curative resection of the localized colorectal cancer, 36 (57.1%) progressed during or within 6 months after the end of adjuvant chemotherapy. For these cases, the adjuvant chemotherapy regimen was considered as the first-line treatment. Among them, five were treated with encorafenib/cetuximab +/− binimetinib in the second-line setting.

The first-line treatments prescribed in the overall population were as follows: FOLFOX/FOLFIRI +/− bevacizumab in 49.2% of cases (*n* = 99), FOLFIRINOX/FOLFOXIRI +/− bevacizumab in 32.8% of cases (*n* = 66), FOLFOX/FOLFIRI + anti-EGFR in 2.5% of cases (*n* = 5), FOLFIRINOX/FOLFOXIRI + anti-EGFR in 3.0% of cases (*n* = 6), fluoropyrimidine +/− bevacizumab in 5.0% of cases (*n* = 10), encorafenib/cetuximab +/− binimetinib in 4.0% of cases (*n* = 8), immune checkpoint inhibitor (ICI) in 1.5% of cases (*n* = 3), and another regimen in 2.0% of cases (*n* = 4).

As described above, encorafenib/cetuximab +/− binimetinib was prescribed in the first line in 4% of cases, in the second line in 56.2% of cases (*n* = 113), in the third line in 28.9% of cases (*n* = 58), and beyond the third line in 10.9% of cases (*n* = 22). Patients were mostly previously exposed to oxaliplatin (81.6%), irinotecan (71.1%), and bevacizumab/aflibercept (71.1%). The majority of patients (79.7%) were in good general condition [Eastern Cooperative Oncology Group performance status (ECOG-PS) 0-1] at the start of treatment. Cetuximab was administered weekly in 61.2% of cases (Table 1). Altogether, 46.3% of patients received at least one subsequent line of treatment after encorafenib/cetuximab +/− binimetinib (55.7% of patients with the targeted therapy prescribed in the second line and 39.6% of patients with the targeted therapy in the third line), with the majority of subsequent first-line therapy being oxaliplatin- and/or irinotecan-based chemotherapy (68% and 65% after the targeted therapy in the second and third line, respectively) (Supplementary Figure S1, available at https://doi.org/10.1016/j.esmoop.2024.103696).

### Treatment tolerability

Table 2 shows the rates of the highest grade of AEs under encorafenib/cetuximab or encorafenib/cetuximab/binimetinib. Overall, 21.7% of patients (*n* = 38) treated with encorafenib/cetuximab and 14.3% of patients (*n* = 3) treated with the triplet encorafenib/cetuximab/binimetinib experienced grade ≥3 AEs (*P* = 0.13).

**Table 2:** Highest grade of adverse events

		Encorafenib/cetuximab (*N* = 180)	Encorafenib/cetuximab/binimetinib (*n* = 21)	*P*			Encorafenib/cetuximab (*N* = 180)	Encorafenib/cetuximab/binimetinib (*n* = 21)	*P*
**All adverse events**	All grades, *n* (%)	159 (90.9)	21 (100)	0.13	**Myalgia**	All grades, *n* (%)	29 (17.7)	4 (20)	0.5
	Grade 3-4, *n* (%)	38 (21.7)	3 (14.3)			Grade 3-4, *n* (%)	3 (1.8)	0 (0)	
	Missing, *n*	5	0			Missing, *n*	16	1	
**Diarrhea**	All grades, *n* (%)	33 (19.9)	14 (66.7)	<0.001∗	**Abdominal pain**	All grades, *n* (%)	38 (23)	7 (33.3)	0.1
	Grade 3-4, *n* (%)	1 (0.6)	2 (9.5)			Grade 3-4, *n* (%)	6 (3.6)	0 (0)	
	Missing, *n*	14	0			Missing, *n*	15	0	
**Constipation**	All grades, *n* (%)	19 (11.7)	3 (15)	0.6	**Headache**	All grades, *n* (%)	8 (5.0)	1 (5)	0.4
	Grade 3-4, *n* (%)	1 (0.6)	0 (0)			Grade 3-4, *n* (%)	0 (0)	0 (0)	
	Missing, *n*	17	1			Missing, *n*	19	1	
**Nausea/vomiting**	All grades, *n* (%)	40 (24.2)	5 (25)	0.9	**Anemia**	All grades, *n* (%)	46 (27.5)	4 (20)	0.8
	Grade 3-4, *n* (%)	5 (3.0)	0 (0)			Grade 3-4, *n* (%)	6 (3.6)	0 (0)	
	Missing, *n*	15	1			Missing, *n*	13	1	
**Mucositis**	All grades, *n* (%)	21 (12.7)	4 (19)	0.4	**Thrombopenia**	All grades, *n* (%)	3 (1.8)	0 (0)	>0.99
	Grade 3-4, *n* (%)	3 (1.8)	0 (0)			Grade 3-4, *n* (%)	0 (0)	0 (0)	
	Missing, *n*	15	0			Missing, *n*	16	1	
**Acneiform dermatitis/rash**	All grades, *n* (%)	78 (45.9)	18 (85.7)	<0.001∗	**Neutropenia**	All grades, *n* (%)	7 (4.3)	0 (0)	>0.99
	Grade 3-4, *n* (%)	1 (0.6)	1 (4.8)			Grade 3-4, *n* (%)	0 (0)	0 (0)	
	Missing, *n*	10	0			Missing, *n*	16	1	
**Pruritus**	All grades, *n* (%)	25 (15.2)	4 (20)	0.6	**AST/ALT increase**	All grades, *n* (%)	20 (12.3)	0 (0)	0.7
	Grade 3-4, *n* (%)	0 (0)	0 (0)			Grade 3-4, *n* (%)	4 (2.4)	0 (0)	
	Missing, *n*	16	1			Missing, *n*	17	1	
**Skin dryness**	All grades, *n* (%)	52 (31.9)	8 (40)	0.06	**Bilirubin increase**	All grades, *n* (%)	10 (6.1)	0 (0)	>0.99
	Grade 3-4, *n* (%)	0	0 (0)			Grade 3-4, *n* (%)	2 (1.2)	0 (0)	
	Missing, *n*	17	1			Missing, *n*	16	1	
**Hand-foot syndrome**	All grades, *n* (%)	8 (4.9)	1 (4.8)	0.7	**Creatinine increase**	All grades, *n* (%)	4 (2.4)	4 (20)	0.001∗
	Grade 3-4, *n* (%)	1 (0.6)	0 (0)			Grade 3-4, *n* (%)	3 (1.8)	0 (0)	
	Missing, *n*	16	0			Missing, *n*	17	1	
**Blurred vision**	All grades, *n* (%)	5 (3.1)	3 (14.3)	**0.01**∗	**Febrile neutropenia**	Yes	1 (0.6)	0 (0)	>0.99
	Grade 3-4, *n* (%)	0 (0)	0 (0)			Missing (*n*)	9	1	
	Missing, *n*	21	0		**Keratoacanthoma and/or SCC**	Yes	5 (2.9)	0 (0)	>0.99
**Peripheral oedema**	All grades, *n* (%)	7 (4.3)	1 (4.8)	0.3		Missing (*n*)	7	0	
	Grade 3-4, *n* (%)	0	0 (0)		**Skin BCC**	Yes	1 (0.6)	0 (0)	>0.99
	Missing, *n*	17	0			Missing (*n*)	5	0	
**Arthralgia**	All grades, *n* (%)	42 (25)	2 (9.5)	0.3	**Melanocytic nevi**	Yes	18 (10.3)	0 (0)	0.2
	Grade 3-4, *n* (%)	4 (2.4)	0 (0)			Missing (*n*)	6	0	
	Missing, *n*	12	0						

ALT, alanine transaminase; AST, aspartate transaminase; BCC, basal cell carcinoma; SCC, squamous cell carcinoma.

∗ *P* < 0.05.

In patients treated with encorafenib/cetuximab, all grade ≥3 AEs occurred in <5% of cases, with the more common ones being arthralgia/myalgia (3.9%), abdominal pain (3.6%), anemia (3.6%), nausea/vomiting (3%), and aspartate transaminase (AST)/alanine transaminase (ALT) increase (2.4%). The most frequent grade 1-2 AEs under encorafenib/cetuximab were acneiform dermatitis/rash (42.7%), skin dryness (31.9%), arthralgia/myalgia (35.5%), and anemia (22.2%). No toxic deaths were observed.

All-grade diarrhea (66.7% with the triplet regimen versus 19.9% with the doublet regimen, *P* < 0.001), acneiform dermatitis/rash (85.7% versus 45.9%, respectively, *P* < 0.001), blurred vision (14.3% versus 3.1%, respectively, *P* = 0.01), and creatinine increase (20.0% versus 2.4%, respectively, *P* = 0.001) were significantly more frequent in patients treated with the triplet encorafenib/cetuximab/binimetinib compared with those treated with the doublet encorafenib/cetuximab.

Cyclins were prescribed at the beginning of treatment in 53.0% of cases as primary prophylaxis for skin toxicity.

Concerning the incidence of skin tumors under treatment, 5 patients experienced keratoacanthoma and/or squamous cell carcinoma during treatment, 1 patient had a basal cell carcinoma, and 18 patients (10.3%) had melanocytic nevi. These 24 patients were all treated with the doublet encorafenib/cetuximab.

Regarding the tolerance according to the line of therapy, the rate of grade 3-4 AEs was numerically higher in patients treated with encorafenib/cetuximab +/− binimetinib from the third line compared with those treated in the first or second line (25.6% versus 17.9%, respectively, *P* = 0.2).

### Treatment adherence of encorafenib cetuximab (n = 180)

The dose of encorafenib was reduced in 36 patients (20.0%; with a dosage of 225 mg in 11.7% of cases, 150 mg in 6.1% of cases, and another dosage in 2.2% of cases, and the dose of cetuximab was reduced in 7 patients (3.9%; with a reduction of –25% in 2 patients, −50% in 4 patients, and another adjustment dosage in 1 patient).

The treatment was discontinued due to encorafenib-related toxicity in six patients (3.3%) and cetuximab-related toxicity in seven patients (4.1%). Among these last patients, two had an allergic reaction to cetuximab and were subsequently switched to panitumumab in combination with encorafenib.

### Best response rates

Among the 177 patients under encorafenib/cetuximab +/− binimetinib with a tumor evaluation, the DCR was 71.2%, including 1.1% of complete response, 31.1% of partial response, and 39.0% of stable disease. Progressive disease as the best response was observed in 28.8% of patients. There was no significant difference in the ORR between encorafenib/cetuximab and encorafenib/cetuximab/binimetinib, but the DCR was numerically higher in the encorafenib/cetuximab/binimetinib group (85.7% versus 69.2%, *P* = 0.06; Table 3). According to lines, the ORR and DCR were also numerically higher in patients treated in the first or second line compared with those treated beyond the second line (ORR: 35.6% versus 27.8%, respectively, *P* = 0.28 and DCR: 75% versus 66.7%, respectively, *P* = 0.23).

**Table 3:** Best response rates and survival under encorafenib/cetuximab +/− binimetinib

Best response rates				
	Whole population (*N* = 201), *n* (%)	Encorafenib/cetuximab (*n* = 180), *n* (%)	Encorafenib/cetuximab/binimetinib (*n* = 21), *n* (%)	*P* value
**Complete response**	2 (1.1)	1 (0.6)	1 (4.8)	0.065
**Partial response**	55 (31.1)	49 (31.4)	6 (28.6)	
**Objective response**	57 (32.2)	50 (32.0)	7 (33.3)	
**Stable disease**	69 (39.0)	58 (37.2)	11 (52.4)	
**Disease control**	126 (71.2)	108 (69.2)	18 (85.7)	
**Progressive disease**	51 (28.8)	48 (30.8)	3 (14.3)	
**No assessable (*n*)**	20	20	0	
**Missing (*n*)**	4	4	0	

**Survival in the whole population (*N* = 201)**								
	PFS				OS			
	Patients, *n*	Events, *n*	Median (95% CI)	*P*	Patients, *n*	Events, *n*	Median (95% CI)	*P*
**All lines**	197	176	4.5 (3.9-5.4)		198	162	9.1 (7.8-10.8)	
**Line 1 or line 2**	118	108	4.8 (3.9-5.8)	0.7	120	95	10.5 (8.7-13.4)	0.03∗
**Line 2**	110	100	4.8 (3.9-5.8)		112	88	10.2 (8.6-13.2)	
**Beyond line 2**	79	68	4.1 (3.6-5.8)		78	67	7.6 (5.9-10.1)	

CI, confidence interval; OS, overall survival; PFS, progression-free survival.

∗ *P* < 0.05.

Twelve patients under encorafenib/cetuximab (6.7%) and none under encorafenib/cetuximab/binimetinib underwent a locoregional treatment (surgery of metastases in six patients and stereotactic radiotherapy in six patients) mainly in the second-line setting (9/12).

In the 23 patients with an MSI-high and/or dMMR tumor, 12 patients (52.2%) received the sequence ICI (all lines) immediately followed by encorafenib/cetuximab +/− binimetinib with an ORR under encorafenib/cetuximab +/− binimetinib of 30.8% (*n* = 4) and a DCR of 53.8% (*n* = 7), 6 patients received encorafenib/cetuximab +/− binimetinib with an interval treatment after ICI, and 5 patients (21.7%) never received ICI after encorafenib/cetuximab +/− binimetinib (because of death or other reasons).

### Survival

**Figure 1:**
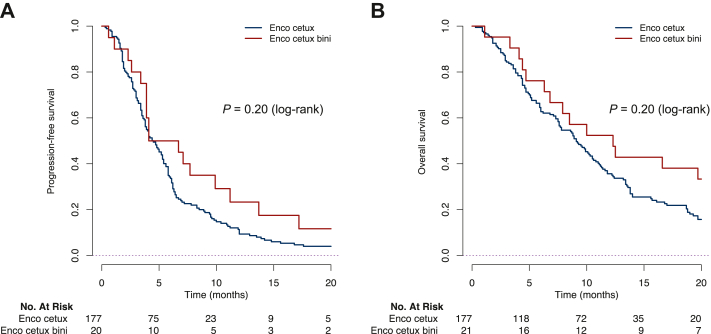
**Kaplan–Meier curves for progression-free survival and overall survival in patients treated with encorafenib/cetuximab +/− binimetinib.** (A) Progression-free survival in patients treated with encorafenib + cetuximab and in those treated with encorafenib + cetuximab + binimetinib. (B) Overall survival in patients treated with encorafenib + cetuximab and in those treated with encorafenib + cetuximab + binimetinib. Bini, binimetinib; Cetux, cetuximab; Enco, encorafenib.

After a median follow-up of 37.3 months (95% CI 30.7-41.9 months) from the start of encorafenib/cetuximab +/− binimetinib, 176 (87.6%) patients had progressed, and 162 (80.6%) patients died. In the overall population treated with encorafenib/cetuximab +/− binimetinib, the median PFS was 4.5 months (95% CI 3.9-5.4 months) across all lines without significant difference according to treatment line. The median OS was 9.2 months (95% CI 7.8-10.8 months; Table 3). The median PFS and OS were 4.5 months (95% CI 3.8-5.4 months) and 9.1 months (95% CI 7.6-10.6 months), respectively, in the 176 patients treated with the doublet encorafenib/cetuximab and 5.4 months (95% CI 3.9-13.7 months) and 12.3 months (95% CI 6.8-28.3 months), respectively, in the 21 patients treated with the triplet encorafenib/cetuximab/binimetinib (*P* = 0.20 and *P* = 0.20 or PFS and OS, respectively; Figure 1).

In univariable analyses, factors associated with a shorter PFS under encorafenib/cetuximab/binimetinib were synchronous metastases [hazard ratio (HR) 1.54, 95% CI 1.05-2.25, *P* = 0.03], liver metastases (HR 1.52, 95% CI 1.12-2.06, *P* = 0.007), baseline ECOG-PS > 1 (HR 1.67, 95% CI 1.15-2.41, *P* = 0.007), and factors associated with a shorter OS were synchronous metastases (HR 1.53, 95% CI 1.02-2.29, *P* = 0.04), treatment line beyond the second line (HR 1.40, 95% CI 1.02-1.92, *P* = 0.03), baseline ECOG-PS >1 (HR 2.11, 95% CI 1.44-3.08, *P* < 0.001), and baseline CEA >16.4 UI/mL (median; HR 1.77, 95% CI 1.25-2.51, *P* = 0.001). Similar PFS and OS rates were observed in patients receiving cetuximab every week or every 2 weeks (Table 4).

**Table 4:** Factors associated with PFS and OS in univariate and multivariable analyses in patients treated with encorafenib/cetuximab +/− binimetinib (*N* = 201)

Factors		PFS						OS					
		Univariate analyses			Multivariable model			Univariate analyses			Multivariable model		
		HR	95% CI	*P*	HR	95% CI	*P*	HR	95% CI	*P*	HR	95% CI	*P*
**Male sex**		0.98	0.73-1.32	0.90	—	—	—	0.99	0.73-1.35	0.96	—	—	—
**Age >65 years**		0.85	0.63-1.15	0.29	—	—	—	1.06	0.78-1.45	0.70	—	—	—
**Surgery of the primary tumor**		0.77	0.55-1.07	0.12	—	—	—	0.84	0.60-1.18	0.32	—	—	—
**Primary tumor location**	**Left sided**	0.95	0.68-1.35	0.3	—	—	—	0.97	0.68-1.39	0.79	—	—	—
	**Rectum**	1.40	0.88-2.23	—	—	—	—	1.16	0.72-1.86	—	—	—	—
**Synchronous metastases**		1.54	1.05-2.25	0.03∗	1.66	1.02-2.72	0.04∗	1.53	1.02-2.29	0.04∗	1.71	1.07-2.74	0.03∗
**Liver metastases**		1.52	1.12-2.06	0.007∗	1.17	0.79-1.72	0.4	1.10	0.80-1.50	0.56	—	—	—
**Pulmonary metastases**		1.11	0.77-1.59	0.57	—	—	—	1.25	0.87-1.81	0.23	—	—	—
**Peritoneal metastases**		0.84	0.63-1.14	0.26	—	—	—	0.83	0.61-1.14	0.25	—	—	—
**Lymph node metastases**		1.08	0.76-1.53	0.66	—	—	—	0.88	0.61-1.26	0.47	—	—	—
**Number of metastatic sites >3**		1.85	0.94-3.64	0.077	1.53	0.69-3.40	0.3	1.09	0.53-2.22	0.81	—	—	—
**pMMR status**		1.01	0.61-1.68	0.97	—	—	—	0.86	0.51-1.43	0.56	—	—	—
**Line encorafenib/cetuximab +/− binimetinib > line 2**		1.05	0.77-1.42	0.75	1.16	0.81-1.67	0.4	1.40	1.02-1.92	0.04∗	1.74	1.21-2.51	0.003∗
**Baseline CEA > 16.4 UI/mL (median)**		1.39	0.98-1.96	0.065	1.37	0.95-1.96	0.09	1.77	1.25-2.51	0.001∗	1.72	1.21-2.44	0.003∗
**ECOG-PS >1**		1.67	1.15-2.41	0.007∗	1.88	1.19-2.99	0.007∗	2.11	1.44-3.08	<0.001∗	2.36	1.51-3.69	<0.001∗
**Cetuximab administration schedule: 2 weeks**		0.86	0.63-1.17	0.34	—	—	—	0.88	0.64-1.21	0.43	—	—	—
**Triplet encorafenib/cetuximab/binimetinib**		0.72	0.44-1.16	0.17	—	—	—	0.71	0.44-1.15	0.17	—	—	—

CEA, carcinoembryonic antigen; CI, confidence interval; ECOG-PS, Eastern Cooperative Oncology Group performance status; HR, hazard ratio; OS, overall survival; PFS, progression-free survival; pMMR, proficient mismatch repair.

∗ *P* values <0.05.

In multivariable analyses, synchronous metastases and baseline ECOG-PS >1 were significantly associated with a shorter PFS (HR 1.66, 95% CI 1.02-2.72, *P* = 0.04 and HR 1.88, 95% CI 1.19-2.99, *P* = 0.007, respectively), whereas synchronous metastases, treatment beyond the second line, baseline ECOG-PS >1, and baseline CEA >16.4 UI/ml (median) were significantly associated with a shorter OS (Table 4).

PFS and OS were not significantly different between anti-EGFR-naive patients and the 24 patients preexposed to anti-EGFR therapy (median PFS 4.5 months, 95% CI 3.9-5.6 months and median PFS 3.9 months, 95% CI 3.4-5.8 months, respectively, *P* = 0.05 and median OS 9.5 months, 95% CI 7.8-11.3 months and median OS 8.8 months, 95% CI 6.8-13.8 months, respectively, *P* = 0.5), nor were the response rates (Supplementary Tables S1 and S2, available at https://doi.org/10.1016/j.esmoop.2024.103696).

## Discussion

This real-world study in patients with *BRAF* V600E-mutated mCRC treated with encorafenib/cetuximab +/− binimetinib shows very similar efficacy and safety data as compared with the BEACON CRC registration trial.

The characteristics of our study population are typical of patients with *BRAF* V600E-mutated mCRC, with a majority of women, a predominance of right-sided tumors, and the most frequent metastatic sites being the liver and peritoneum.^17^ However, the proportion of patients with MSI-high/dMMR phenotype is underrepresented, comprising only 11% of the population instead of the 20%-35% described in the literature,^18^^,^^19^ likely due in part to patients with long-lasting disease control with ICI treatment, before considering treatment with encorafenib and cetuximab. Indeed, these patients with MSI-high/dMMR tumors have access to ICI (pembrolizumab) as a standard first-line treatment since the publication of the Keynote-177 trial.^20^ In addition, in these patients, the *BRAF* V600E mutation did not appear to be a factor of resistance to ICI.^20^^,^^21^

This real-world study population has the advantage of providing data on the efficacy and tolerability of encorafenib/cetuximab +/− binimetinib in patients with ECOG-PS >1 (20%) and beyond the third line (11%), who were excluded from the BEACON CRC trial. In addition, in 39% of cases, cetuximab was administered every 2 weeks at a dose of 500 mg/m^2^, despite the approved dosing regimen being an initial dose of 400 mg/m^2^ followed by 250 mg/m^2^ weekly, as outlined in the BEACON CRC protocol when used in conjunction with encorafenib +/– binimetinib.^11^ The biweekly regimen was not associated with a decrease in PFS or OS in our population and has the advantage of extending the intervals between the patient’s visits to the day hospital. In this regard, it has been demonstrated that administering cetuximab at 500 mg/m^2^ every 2 weeks is equivalent to cetuximab at 250 mg/m^2^ weekly in terms of pharmacokinetic and pharmacodynamic parameters, and especially in terms of tolerance and efficacy, which is becoming a convenient alternative.^22^, ^23^, ^24^ In addition, biweekly administration of cetuximab is used in the ongoing BREAKWATER trial evaluating chemotherapy + encorafenib + cetuximab in *BRAF* V600E-mutant mCRC in the first-line setting.^25^

Regarding tolerability, subject to the retrospective nature of the study, which may limit the quality of AE reporting, the tolerance profile was generally favorable in our study population, with only 21% experiencing AEs of grade ≥3, with the most common being under encorafenib/cetuximab: arthralgia/myalgia, abdominal pain, nausea/vomiting, anemia, and AST/ALT increase, with incidence rates consistently below 5%, and numerically lower rate of ≥3 AEs in patients treated with encorafenib/cetuximab +/− binimetinib in first- or second-line compared with those treated beyond the second line. The most frequent grade 1-2 AEs under encorafenib/cetuximab were acneiform dermatitis/rash, skin dryness, arthralgia/myalgia, and anemia, which is broadly consistent with the findings in the BEACON CRC trial and other real-world studies reporting on the encorafenib/cetuximab combination.^11^^,^^13^^,^^26^ However, compared with the BEACON CRC trial, the rate of dermatological toxicities is lower in our study population (50% versus 75%), probably partly due to the retrospective nature of our study, resulting in an underreporting of low-grade toxicities. It may be also partly attributed to the prescription of primary prophylaxis with a cyclin in 53% of cases; however, this was not mandatory in the BEACON CRC protocol.^11^ Compared with the doublet encorafenib/cetuximab, the triplet encorafenib/cetuximab/binimetinib was associated with a numerically lower rate of grade 3-4 AEs, in contrast to the BEACON CRC trial. This can be partly explained by the small number of patients treated with the triplet and/or the prescription of the triplet to patients in better overall condition. However, patients treated with the triplet experienced more all-grade diarrhea, acneiform dermatitis/rash, blurred vision, and creatinine increase, which was also observed in the BEACON CRC trial.^11^^,^^27^ Notably, 20% of our patients treated with the triplet had creatinine increase (all grades) compared with only 2.4% in the doublet, which may be a specific side-effect of the mitogen-activated protein kinase kinase (MEK) inhibitors class.^28^ Interestingly, as observed in the BEACON CRC trial, the occurrence of skin tumors (melanocytic naevi, keratoacanthoma, squamous cell carcinoma, or basal cell carcinoma) under treatment was present only with the doublet encorafenib/cetuximab and not in any of the patients treated with the triplet.^27^ It suggests, as previously reported, that the MEK inhibitor may have a protective effect on the incidence of these skin tumors.^29^ The addition of an MEK inhibitor to a BRAF inhibitor has been shown, in patients with melanoma, to limit the paradoxical activation of the ERK pathway and subsequent skin cell proliferation.^29^^,^^30^ Because of this good overall tolerability of the treatment, a minority of patients had a reduction in treatment dose (20% for encorafenib) or AE-related treatment discontinuation (7%), as observed in other cohorts in a real-world setting.^13^

Our study population is not strictly comparable to that of the BEACON registration trial due to the presence of better prognostic factors: 4% of patients were treated in the first line, only 20% had three or more metastatic sites (compared with 47% and 49% in the encorafenib/cetuximab or encorafenib/cetuximab/binimetinib groups of the BEACON population, respectively), and 82% had prior exposure to oxaliplatin (compared with 89% and 95%, respectively). However, our population also included patients with poorer prognosis factors, such as 20% with an ECOG-PS >1 and 11% treated beyond the third line. Despite these factors, we observed similar efficacy outcomes: an ORR of 32.2% (compared with 20% and 26%, respectively, in the BEACON trial), a DCR of 71.2% (compared with 74% and 80%, respectively), a median PFS of 4.5 months (compared with 4.2 and 4.3 months, respectively) and a median OS of 9.2 months (compared with 8.4 and 9 months, respectively) in the overall population treated with encorafenib/cetuximab +/− binimetinib.^11^ The median PFS was also similar to that reported in the two other real-world studies by Fernandez Montes et al.^14^ and Boccaccino et al.^13^ (5 and 4.5 months, respectively). By contrast, the median OS was slightly higher in the Spanish cohort (12.6 months), likely because all patients received the treatment in second line,^14^ while the median OS in the Italian cohort was slightly lower (7.2 months), probably due to a higher proportion of patients with an ECOG-PS >1 (34%).^13^

ORR and DCR were numerically higher in patients treated earlier in the first or second line, compared with those treated beyond the second line. However, we observed no significant difference for PFS according to line, likely in part because this is a therapy targeting a driver mutation.

According to the treatment regimen, we observed a trend toward a better DCR and improved OS with the triplet regimen, although this did not reach statistical significance (DCR: 85.7% versus 69.2%, *P* = 0.06; median OS: 12.3 months versus 9.1 months with the doublet, *P* = 0.20). This lack of significance is likely due, in part, to the small sample size of the triplet subgroup (*n* = 21), which limited the statistical power of the analysis. The real-world study by Boccaccino et al. also observed a similar trend toward a better DCR with the triplet regimen.^13^ It has been hypothesized that a subgroup of patients with a worse prognosis may benefit from the addition of an MEK inhibitor, particularly those with a higher BRAF-mutant allele fraction in circulating tumor DNA^31^ or tumor tissue,^13^ or those classified under the CMS4 or BM1 subgroups based on transcriptome analysis.^32^^,^^33^ Interestingly, the efficacy of the encorafenib/cetuximab +/– binimetinib combination was not impacted by prior administration of anti-EGFR, as previously reported by Hafliger et al.^34^

In multivariable analyses, the factors independently associated with a shorter PFS were synchronous metastases and poor general condition with an ECOG-PS > 1. Similarly, the factors independently associated with a shorter OS were also these two factors, along with the prescription of encorafenib/cetuximab +/− binimetinib beyond the second line of treatment and a high baseline CEA plasma level. This prognostic value of baseline ECOG-PS on both PFS and OS in multivariable analysis was also demonstrated in a real-world study by Boccaccino et al.^13^

Finally, we confirmed the findings from the real-world Italian study of Germani et al.^35^ on attrition rates, showing that 55.7% of patients received at least one subsequent line of therapy when treated in the second line, compared with only 39.6% when treated in the third line, with irinotecan- and oxaliplatin-based treatments being the preferred options after this targeted therapy. The study also indicated a longer postprogression survival in patients treated earlier in the second line, suggesting that initiating this new combination as soon as possible after the failure of first-line treatment may be beneficial.^35^

The most important strengths of our study are that it is the largest real-world study to date involving patients with *BRAF* V600E mCRC treated with encorafenib/cetuximab +/− binimetinib, and it has the longest follow-up duration for this recently approved treatment.^13^^,^^14^^,^^36^ However, our work also has some limitations. Its retrospective nature and the small number of patients in various treatment subgroups restrict the ability to carry out meaningful subgroup analyses, particularly for the triplet regimen of encorafenib/cetuximab/binimetinib or the MSI-high subgroup. Additionally, because 91% of patients were treated in specialized and/or academic centers, which often offer more treatment options, this may lead to earlier use of this targeted therapy. Consequently, these results may not be fully generalizable to patients treated in other types of centers.

To conclude, this real-world multicentric study in patients with *BRAF* V600E mCRC treated with encorafenib/cetuximab +/− binimetinib confirms the efficacy and safety outcomes observed in the BEACON CRC registration trial. Poor general condition, synchronous metastases, high CEA level, and two or more prior lines of therapy were identified as the main prognostic factors under this targeted therapy. These data, combined with the high attrition rate in this specific population of *BRAF* V660E-mutated patients, suggest that the optimal timing for this new targeted therapy may be as early as possible after progression on first-line treatment. The results of the ongoing phase III BREAKWATER trial are awaited to determine whether an earlier administration in the first line, in combination with chemotherapy, would provide additional benefits for these patients.^25^
