# Supplementary material for: Efficacy and safety of the combination of encorafenib/cetuximab with or without binimetinib in patients with BRAF V600E-mutated metastatic colorectal cancer: an AGEO real-world multicenter study
Source: ESMO Open. 2024 Sep 9;9(9):103696. doi: 10.1016/j.esmoop.2024.103696 (PMC11415680; doi:10.1016/j.esmoop.2024.103696)
Supplement: Supplementary data [file mmc1.docx]

**Supplementary Materials**

**Supplemental Figure 1. Subsequent lines after progression to encorafenib + cetuximab +/-binimetinib in the overall population and in patients treated with encorafenib + cetuximab +/- binimetinib in 2^nd^ line and 3^rd^ line**

A. Subsequent lines after progression to encorafenib + cetuximab +/- binimetinib in the overall population. B. Subsequent lines after progression to encorafenib + cetuximab +/- binimetinib prescribed in 2^nd^ line. B. Subsequent lines after progression to encorafenib + cetuximab +/- binimetinib prescribed in 3^rd^ line.

**Supplemental Table 1. Survival endpoints according to the prior exposure to anti-EGFR therapies in patients treated with encorafenib, cetuximab +/- binimetinib (N=201)**

|  | N patients | N events | Median (months) (95%CI) | log-rank p |
| --- | --- | --- | --- | --- |
| **Overall survival** | | | | |
| Pre-exposed to anti-EGFR | 25 | 21 | 8.8 (6.8-13.8) | 0.5 |
| Naive | 173 | 141 | 9.5 (7.8-11.3) |  |
| **Progression -free survival** | | | | |
| Pre-exposed to anti-EGFR | 25 | 23 | 3.9 (3.4-5.8) | 0.05 |
| Naive | 172 | 153 | 4.5 (3.9-5.6) |  |

95%CI: 95% confidence interval

**Supplemental Table 2. Response rates according to the prior exposure to anti-EGFR therapies in patients treated with encorafenib, cetuximab +/- binimetinib (N=201)**

|  |  | Overall population | anti-EGFR naive | Pre-exposed to anti EGFR | p-value |
| --- | --- | --- | --- | --- | --- |
|  |  | (N=201) | (N=176) | (N=25) |  |
| **Objective response** | | 57 (32.2%) | 47 (30.5%) | 10 (43.5%) | 0.21 |
|  | Missing | 24 | 22 | 2 |  |
| **Disease control** | | 126 (71.2%) | 110 (71.4%) | 16 (69.6%) | 0.85 |
|  | Missing | 24 | 22 | 2 |  |
